# Sex-related differences in oncologic outcomes, operative complications and health-related quality of life after curative-intent oesophageal cancer treatment: multicentre retrospective analysis

**DOI:** 10.1093/bjsopen/zrae026

**Published:** 2024-04-03

**Authors:** Styliani Mantziari, Jessie A Elliott, Sheraz R Markar, Fredrik Klevebro, Lucas Goense, Asif Johar, Pernilla Lagergren, Giovanni Zaninotto, Richard van Hillegersberg, Mark I van Berge Henegouwen, Markus Schäfer, Magnus Nilsson, George B Hanna, John V Reynolds, ENSURE Study Group, Hans Van Veer, Lieven Depypere, Willy Coosemans, Philippe Nafteux, Paul Carroll, Frances Allison, Gail Darling, John M Findlay, Serenydd Everden, Nicholas D Maynard, Arun Ariyarathenam, Grant Sanders, Shameen Jaunoo, Pritam Singh, Simon Parsons, John Saunders, Ravinder Vohra, Aaditya Sinha, Benjamin H L Tan, John G Whiting, Piers R Boshier, Sheraz R Markar, Giovanni Zaninotto, George B Hanna, Alexander W Phillips, S Michael Griffin, Robert C Walker, Tim J Underwood, Guillaume Piessen, Jorg Theisen, Hans Friess, Christiane J Bruns, Wolfgang Schröder, Chris G Collins, Oliver J McAnena, Siobhan Rooney, Aoife Quinn, Conor Toale, Thomas J Murphy, Jessie A Elliott, Narayanasamy Ravi, Claire L Donohoe, John V Reynolds, Marco Scarpa, Romeo Bardini, Silvia Degasperi, Luca Saadeh, Carlo Castoro, Rita Alfieri, Eleonora Pinto, Genny Mattara, Marianne C Kalff, Suzanne S Gisbertz, Mark I van Berge Henegouwen, Sander J M van Hootegem, Sjoerd M Lagarde, B Feike Kingma, Lucas Goense, Jelle P Ruurda, Richard van Hillegersberg, Raymond Kennedy, P Declan Carey, Leanne Prodehl, Peter J Lamb, Richard J E Skipworth, Mariagiulia Dal Cero, Manuel Pera, Biying Huang, Fredrik Klevebro, Magnus Nilsson, Asif Johar, Pernilla Lagergren, Gustav Linder, Magnus Sundbom, Styliani Mantziari, Markus Schäfer, Nicolas Demartines

**Affiliations:** Department of Visceral Surgery, Lausanne University Hospital CHUV, Lausanne, Switzerland; Faculty of Biology and Medicine, University of Lausanne UNIL, Lausanne, Switzerland; Trinity St. James’s Cancer Institute, Trinity College Dublin, and St. James’s Hospital, Dublin, Ireland; Surgical Intervention Trials Unit, Nuffield Department of Surgical Sciences, University of Oxford, Oxford, UK; Karolinska Institutet, Department of Molecular Medicine and Surgery, Karolinska University Hospital, Stockholm, Sweden; CLINTEC, Karolinska Institutet, Stockholm, Sweden; Department of Surgery, University Medical Center Utrecht, Utrecht University, Utrecht, The Netherlands; Karolinska Institutet, Department of Molecular Medicine and Surgery, Karolinska University Hospital, Stockholm, Sweden; Karolinska Institutet, Department of Molecular Medicine and Surgery, Karolinska University Hospital, Stockholm, Sweden; CLINTEC, Karolinska Institutet, Stockholm, Sweden; Department of Surgery and Cancer, Imperial College, London, UK; Department of Surgery, University Medical Center Utrecht, Utrecht University, Utrecht, The Netherlands; Department of Surgery, Amsterdam UMC, University of Amsterdam, Amsterdam, The Netherlands; Cancer Center Amsterdam, Amsterdam, The Netherlands; Department of Visceral Surgery, Lausanne University Hospital CHUV, Lausanne, Switzerland; Faculty of Biology and Medicine, University of Lausanne UNIL, Lausanne, Switzerland; Karolinska Institutet, Department of Molecular Medicine and Surgery, Karolinska University Hospital, Stockholm, Sweden; CLINTEC, Karolinska Institutet, Stockholm, Sweden; Department of Surgery and Cancer, Imperial College, London, UK; Trinity St. James’s Cancer Institute, Trinity College Dublin, and St. James’s Hospital, Dublin, Ireland

## Abstract

**Background:**

Oesophageal cancer, in particular adenocarcinoma, has a strong male predominance. However, the impact of patient sex on operative and oncologic outcomes and recovery of health-related quality of life is poorly documented, and was the focus of this large multicentre cohort study.

**Methods:**

All consecutive patients who underwent oncological oesophagectomy from 2009 to 2015 in the 20 European iNvestigation of SUrveillance after Resection for Esophageal cancer study group centres were assessed. Clinicopathologic variables, therapeutic approach, postoperative complications, survival and health-related quality of life data were compared between male and female patients. Multivariable analyses adjusted for age, sex, tumour histology, treatment protocol and major complications. Specific subgroup analyses comparing adenocarcinoma *versus* squamous cell cancer for all key outcomes were performed.

**Results:**

Overall, 3974 patients were analysed, 3083 (77.6%) male and 891 (22.4%) female; adenocarcinoma was predominant in both groups, while squamous cell cancer was observed more commonly in female patients (39.8% *versus* 15.1%, *P* < 0.001). Multivariable analysis demonstrated improved outcomes in female patients for overall survival (HR_males_ 1.24, 95% c.i. 1.07 to 1.44) and disease-free survival (HR_males_ 1.22, 95% c.i. 1.05 to 1.43), which was caused by the adenocarcinoma subgroup, whereas this difference was not confirmed in squamous cell cancer. Male patients presented higher health-related quality of life functional scores but also a higher risk of financial problems, while female patients had lower overall summary scores and more persistent gastrointestinal symptoms.

**Conclusion:**

This study reveals uniquely that female sex is associated with more favourable long-term survival after curative treatment for oesophageal cancer, especially adenocarcinoma, although long-term overall and gastrointestinal health-related quality of life are poorer in women.

## Teaser

In this large series of oesophageal cancer patients treated with curative intent, drawing from the ENSURE (European iNvestigation of SUrveillance after Resection for Esophageal cancer) registry, several key outcomes presented sex-related differences. Female patients, despite higher major morbidity rates after surgery and more long-term gastrointestinal complications, displayed better overall and disease-free survival compared with males.

## Introduction

Oesophageal cancer (OC) has a strong male predominance, accounting for 75–89% of patients in published series^1–3^. Recently, sex-related differences have been suggested in OC cancer biology, in terms of treatment response, toxicity and oncologic outcomes^4,5^. Furthermore, female patients experience higher toxicity from standard doses of systemic chemotherapy than males, which cannot solely be attributed to lean body mass differences^5^. Moreover, among patients with OC of similar stage and tumour location, female patients were reported to receive less often transthoracic oesophagectomy and neoadjuvant therapy^6,7^, or even cancer-specific systemic treatment in case of metastatic/advanced disease^8^.

Sex-related differences have previously been described in gastric and colorectal cancer for instance, where an obvious link between hormonal drive and tumour progression is not known to exist^7,9^. Such results have also been described for OC, where male patients were reported to have poorer long-term survival after surgery^3,6,10^. A Swedish nationwide study reported a prognostic advantage for female patients with oesophageal squamous cell cancer (SCC), whereas no such difference was observed for adenocarcinoma (AC)^11^. In addition, health-related quality of life (HRQoL) is another major long-term issue in OC patients^12^. The LASER (Lasting Symptoms after Esophageal Resection) study revealed that 66.9% of patients have persistent symptoms impacting HRQoL 1 year after oesophagectomy; however, the potential impact of patient sex was not specifically assessed^13,14^. Thus, despite a suggestion of a multifaceted impact of patient sex on OC, the underlying mechanisms and specific impact on key outcome measures remain poorly understood.

The aim of the present study, drawing on a population of almost 4000 patients from the ENSURE (European iNvestigation of SUrveillance after Resection for Esophageal cancer) registry, was to explore potential sex-related differences in treatment strategies, operative and oncologic outcomes, as well as HRQoL after OC treatment with curative intent.

## Methods

### Inclusion criteria

All consecutive patients operated on for OC between January 2009 and June 2015 in the 20 participating European and North-American centres of the ENSURE study group were assessed for eligibility (*Table S1*). Inclusion criteria were age >18 years, treatment with curative intent including surgery, upfront, after neoadjuvant therapy or salvage, for any histological type of non-metastatic (TxNxM0) cancer of the oesophagus or the oesophagogastric junction (Siewert I–III). Patients treated with definitive chemoradiation alone were excluded.

### Study endpoint definitions

Long-term survival was assessed by means of overall survival (OS), disease-specific survival (DSS) and disease-free survival (DFS). Treatment allocation was assessed via the following surrogate endpoints: access to systemic oncological treatment (neoadjuvant, or in case of cancer recurrence) and choice of surgical approach. To assess HRQoL, the EORTC (European Organisation for Research and Treatment of Cancer) QLQ-30 questionnaire^15^ was collected from patients who were recurrence free at 1 year after surgery, through database matching with the LASER dataset^13^.

Postoperative complications were recorded and graded according to the validated 5-scale Clavien–Dindo system. All complications, including mortality rate, are reported for the entire hospital stay after the index operation (in-hospital)^16^. Anastomotic leak and all other specific complication types were universally defined as per the ECCG (Esophagectomy Complications Consensus Group) expert consensus^17^. For HRQoL items, each Likert-scale answer was linearly transformed to a 0–100 scale continuous variable, with a > 10-point difference considered clinically relevant. Higher HRQoL and functional scales illustrate a better functional level, whereas higher symptom scale scores correspond to higher symptom burden. All outcomes of the EORTC QLQ-30 questionnaire, including financial problems, were self-reported^18^.

### Data collection and ethical considerations

Data for the present analysis were prospectively collected within the institutional databases of participating centres. The study was registered in clinicaltrials.gov (NCT03461341) and approved by the primary investigator centre (St James’ Hospital, Dublin, Ireland, approval number #4982, SJH/Tallaght University Hospital Research Ethics Committee #2018-08-CA). All participating centres obtained approval from local institutional review boards, according to local policies (e.g. Vaud Ethics Committee CER-VD #2022-00123). The study was conducted according to the Declaration of Helsinki for medical research.

### Statistical analysis

All relevant clinicopathologic variables were compared between male and female patients. The χ^2^ test was used for categorical variables, while continuous data were compared with the *t*-test and Satterthwaite correction for unequal variances. For time-to-event outcomes, the Kaplan–Meier method and log-rank test were used, while HRQoL variables were expressed with mean scores and standard deviation (s.d.). Multivariable Cox regression was performed for all survival outcomes adjusted for age, ASA class, patient sex, histological type, cN stage, treatment protocol (neoadjuvant treatment, upfront surgery, definitive chemoradiation) and occurrence of major postoperative complications (Clavien ≥ IIIa). For HRQoL continuous outcome variables, multivariable linear regression adjusted for age, sex, histological type, treatment protocol and major postoperative complications (Clavien ≥ IIIa) was performed. Missing data are reported in detail for all studied variables. For multivariable analyses, multiple imputation was performed for missing values.

Planned subgroup analyses for patients with AC and SCC were performed for all selected outcomes (treatment allocation, survival and HRQoL). Two-tailed *P* < 0.05 was the threshold for statistical significance. All statistical analyses were conducted by a dedicated biostatistician (A.J.), using the SAS 9.4 software (SAS Institute Inc., Cary, NC, USA).

## Results

Overall, 3974 patients (3083 (77.6%) male and 891 (22.4%) female) were included in the present study. Baseline characteristics are detailed in *Table 1* for the whole cohort and stratified by sex, and in *Table S2* stratified separately by histological type.

**Table 1:** Baseline patient and tumour characteristics stratified by patient sex

	All *N* = 3974	Male *N* = 3083	Female *N* = 891	*P* value
**ASA class**				0.001
I	1003 (25.2)	740 (24.0)	263 (29.5)	
II	1947 (49.0)	1525 (49.5)	422 (47.4)	
III	861 (21.7)	693 (22.4)	168 (18.9)	
Missing data	163 (4.1)	125 (4.1)	38 (4.2)	
**ECOG performance status**				0.412
0	1370 (34.5)	1069 (34.7)	301 (33.8)	
1	878 (22.1)	687 (22.2)	191 (21.4)	
2	139 (3.5)	101 (3.3)	38 (4.3)	
3	20 (0.5)	17 (0.6)	3 (0.3)	
Missing data	1567 (39.4)	1209 (39.2)	358 (40.2)	
**Histological type**				<0.001
Adenocarcinoma	2845 (71.6)	2389 (77.5)	456 (51.2)	
Squamous cell cancer	822 (20.7)	467 (15.1)	355 (39.8)	
Other	307 (7.7)	227 (7.4)	80 (9)	
**Tumour site**				<0.001
OGJ	1515 (38.1)	1257 (40.8)	258 (29.0)	
Distal oesophagus	1568 (39.4)	1237 (40.1)	331 (37.1)	
Middle third	552 (13.9)	331 (10.7)	221 (24.8)	
Upper third	82 (2.1)	58 (1.9)	24 (2.7)	
Missing data	257 (6.5)	200 (6.5)	57 (6.4)	
**Barrett’s metaplasia**	844 (21.2)	710 (23.0)	134 (15.0)	<0.001
Missing data	892 (22.4)	673 (21.8)	219 (24.6)	
**cT stage**				0.104
0	42 (1.1)	29 (0.9)	13 (1.5)	
1	415 (10.4)	315 (10.2)	100 (11.2)	
2	713 (17.9)	536 (17.4)	177 (19.8)	
3	2202 (55.5)	1737 (56.3)	465 (52.2)	
4	168 (4.2)	135 (4.4)	33 (3.7)	
Missing data	434 (10.9)	331 (10.7)	103 (11.6)	
**cN stage**				<0.001
0	1206 (30.3)	890 (28.9)	316 (35.5)	
1	1501 (37.8)	1185 (38.4)	316 (35.5)	
2	743 (18.8)	606 (19.6)	137 (15.4)	
3	77 (1.9)	58 (1.9)	19 (2.1)	
Missing data	447 (11.2)	344 (11.1)	103(11.6)	
**cM1 stage**	58 (1.5)	46 (1.5)	12 (1.4)	0.736
Missing data	89 (2.2)	73 (2.4)	16 (1.8)	

Values are *n* (%). ECOG, Eastern Cooperative Status Scale; ASA, American Society of Anesthesiologists; OGJ, oesophagogastric junction.

The predominant histological type was AC in both groups; however, the proportion of SCC was significantly higher among female patients (39.8% *versus* 15.1%, *P* < 0.001), who also presented with more middle-third lesions (24.8% *versus* 10.7%, *P* < 0.001) and less extensive lymphatic spread at baseline (cN0 in 35.5% female patients *versus* 28.9% male patients, *P* < 0.001), despite a similar cT stage. Higher rates of cN0 in women were confirmed in AC (39.5% *versus* 30.8% men, *P* = 0.001), but not in the SCC subtype (*Table S2*). R0 resection rates and mean lymph node yield were similar for all patients (*Table 2*). In the whole cohort, more advanced (y)pT and (y)pN stages were observed in male patients, together with a worse pathologic response to neoadjuvant treatment (Tumour Regression Grade (TRG) 4–5 in 22.3% male patients *versus* 18.3% female patients, *P* = 0.006). However, such differences in pathologic stage and tumour regression were not confirmed in a separate analysis of AC and SCC subgroups (*Table S3*).

**Table 2:** Histopathologic characteristics stratified by patient sex

	All *N* = 3974	Male *N* = 3083	Female *N* = 891	*P* value
**(y)pT stage**				<0.001
0	434 (10.9)	298 (9.7)	136 (15.3)	
Tis/high-grade dysplasia	51 (1.3)	35 (1.1)	16 (1.8)	
1	825 (20.8)	641 (20.8)	184 (20.7)	
2	584 (14.7)	455 (14.8)	129 (14.5)	
3	1832 (46.1)	1464 (47.5)	368 (41.3)	
4	189 (4.8)	148 (4.8)	41 (4.6)	
Missing data	59 (1.5)	42 (1.4)	17 (1.9)	
**(y)pN stage**				<0.001
0	1869 (47.0)	1397 (45.3)	472 (53.0)	
1	819 (20.6)	653 (21.2)	166 (18.6)	
2	846 (21.3)	668 (21.7)	178 (20.0)	
3	359 (9.0)	393 (12.7)	56 (6.3)	
Missing data	81 (2.0)	62 (2.0)	19 (2.1)	
**(y)pM1 stage**	90 (2.3)	65 (2.1)	25 (2.8)	0.229
Missing data	38 (0.9)	34 (1.1)	4 (0.4)	
**Differentiation**				0.005
G0	440 (11.1)	315 (10.2)	125 (14.0)	
G1	356 (9.0)	276 (9.0)	80 (9.0)	
G2	1118 (28.1)	878 (28.5)	240 (26.9)	
G3	1091 (27.5)	874 (28.3)	217 (24.4)	
Signet-ring cell	63 (1.6)	53 (1.7)	10 (1.1)	
Missing data	906 (22.8)	687 (22.3)	219 (24.6)	
**Lymphatic invasion (L1)**	1044 (26.3)	841 (27.3)	203 (22.8)	0.005
L0	2091 (52.6)	1592 (51.6)	499 (56.0)	
Missing data	839 (21.1)	650 (21.1)	189 (21.2)	
**Venous invasion (V1)**	1077 (27.1)	862 (27.9)	215 (24.1)	0.024
V0	2273 (57.2)	1740 (56.4)	533 (59.8)	
Missing data	624 (15.7)	481 (15.6)	143 (16.0)	
**Perineural invasion (Pn1)**	730 (18.4)	608 (19.7)	122 (13.7)	<0.001
Pn0	2231 (56.1)	1707 (55.3)	524 (58.8)	
Missing data	1013 (25.5)	768 (24.9)	245 (27.5)	
**Resection margin (R) status**				0.900
R0	3394 (85.4)	2629 (85.3)	765 (85.9)	
R1	517 (13.0)	405 (13.1)	112 (12.6)	
R2	17 (0.4)	13 (0.4)	4 (0.4)	
Missing data	46 (1.2)	36 (1.2)	10 (1.1)	
Mandard regression grade*				0.006
TRG 1	331 (8.3)	242 (7.8)	89 (10.0)	
TRG 2	310 (7.8)	247 (8.0)	63 (7.1)	
TRG 3	354 (8.9)	281 (9.1)	73 (8.2)	
TRG 4	532 (13.4)	429 (13.9)	103 (11.6)	
TRG 5	318 (8.0)	258 (8.4)	60 (6.7)	
Missing data	837 (21.1)	661 (21.4)	176 (19.8)	
Number of lymph nodes involved	2.3(4.2)	2.4(4.4)	1.8(3.6)	<0.001
Number of lymph nodes analysed	26.1(14.0)	26.2(14.1)	25.8(13.8)	0.445

Continuous variables are expressed as mean(s.d.) and categorical variables as *n* (%). *TRG, tumour regression grade (only available in patients that underwent neoadjuvant chemotherapy).

The total hospital stay was longer in female patients (mean(s.d.) 22.7(23) *versus* 20.2(20.9), *P* = 0.005), as was the incidence of major postoperative complications (34.9% female patients *versus* 30.8% male patients, *P* = 0.023). Major morbidity rate did not present significant differences within the two main histological groups (AC, SCC). Overall morbidity rate, the rates of specific complications such as pulmonary complications or anastomotic leakage as well as in-hospital mortality rate did not differ between male and female patients (*Table S4*).

### Influence of patient sex on treatment modalities

#### Choice of treatment strategy

Overall, 58.5% of patients received neoadjuvant treatment (NAT), with similar rates between sexes (*Table S5*). Patient sex was not associated with access to NAT within the whole cohort (adjusted OR 1.18, 95% c.i. 0.78 to 1.43). In the AC group, male patients had increased use of NAT compared with female patients both in univariable and multivariable analysis (adjusted OR 1.28, 95% c.i. 1.01 to 1.62), which was not observed in patients with SCC (adjusted OR 1.05, 95% c.i. 0.73 to 1.52).

In the whole cohort, women more frequently underwent upfront surgery than men (24.7% *versus* 19.3%, *P* = 0.014), whereas rates of definitive chemoradiation (dCRT) and salvage surgery were similar (2% in male patients *versus* 1.5% in female patients). In the AC group, women presented with earlier cN disease stage and they more often received upfront surgery, whereas rates of dCRT were comparable with male patients (1% male *versus* 0.4% female). In the SCC group, where no baseline stage differences were observed, treatment protocol choices were similar, including dCRT rates (2.6% *versus* 1.7% respectively) (*Table S5*). Characteristics of patients treated with upfront surgery and dCRT are presented in *Table S6*, with no significant staging or baseline differences among men and women, apart from higher ASA scores in male patients operated on upfront.

In patients with cancer recurrence, no differences were found in the modalities used for recurrence treatment (radiation in 10.6% male and 9.9% female patients, chemotherapy in 19.1% men and 15.5% women). Patient sex was not independently associated with cancer-specific recurrence treatment in the AC (adjusted OR 1.28, 95% c.i. 0.98 to 1.68) or the SCC subgroups (adjusted OR 0.89, 95% c.i. 0.59 to 1.35).

#### Surgical approach

Ivor Lewis resection was the predominant approach in both groups (49.1% men *versus* 41.9% women, *P* < 0.001) (*Table S5*). Within the AC subtype, female patients had lower rates of Ivor Lewis resection (42.8% *versus* 50.4% in men, *P* < 0.001) despite similar tumour location; such a difference was not observed in patients with SCC (*Table S5*). Open surgery was more frequently performed in female patients (69.7% *versus* 66.3%, *P* = 0.016) (*Table S5*). Indeed, male sex was independently associated with the use of a hybrid minimally invasive approach in the whole cohort (adjusted OR 1.29, 95% c.i. 1.00 to 1.66), as well as the SCC subgroup (adjusted OR 1.93, 95% c.i. 1.08 to 3.44). No difference was observed for the totally minimally invasive approach (adjusted OR 1.02, 95% c.i. 0.80 to 1.30).

### Long-term survival and recurrence patterns

In the whole cohort, median OS was 31.6 months (95% c.i. 12.8 to 58.6) in male *versus* 38.1 months (95% c.i. 15.2 to 60) in female patients (*P* < 0.001), whereas median DFS was 26.7 months (95% c.i. 10.1 to 57.5) and 36.3 months (95% c.i. 12.1 to 60 months) respectively (*P* < 0.001). In multivariable analysis, male sex was associated with poorer long-term OS (HR 1.24, 95% c.i. 1.07 to 1.44) and DFS (HR 1.22, 95% c.i. 1.05 to 1.43), while DSS was similar (HR 1.18, 95% c.i. 0.99 to 1.40) (*Table 3*).

**Table 3:** Multivariable Cox analyses for overall, disease-free and disease-specific survival

	HR _adj_	95% c.i.	*P* value	HR _adj_	95% c.i.	*P* value	HR _adj_	95% c.i.	*P* value
	Overall survival (OS)			Disease-free survival (DFS)			Disease-specific survival (DSS)		
Age (years)	1.01	1.00–1.02	0.002	0.99	0.99–1.00	0.234	1.00	0.99–1.01	0.696
**ASA class**			0.012			0.280			0.408
I	0.846	0.72–1.00		1.00	0.84–1.19		0.97	0.80–1.18	
II	0.813	0.71–0.93		0.91	0.78–1.06		0.90	0.77–1.06	
III	Ref	Ref		Ref	Ref		Ref	Ref	
**Sex**			0.005			0.011			0.054
Male	1.24	1.07–1.44		1.22	1.05–1.43		1.18	0.99–1.40	
Female	Ref	Ref		Ref	Ref		Ref	Ref	
**Histologic type**			0.495			0.163			0.106
AC	1.08	0.92–1.28		1.19	0.99–1.42		1.22	0.99–1.49	
Other	1.15	0.91–1.45		1.21	0.93–1.56		1.30	0.98–1.71	
SCC	Ref	Ref		Ref	Ref		Ref	Ref	
**cN stage**			<0.001			<0.001			<0.001
0	0.41	0.28–0.61		0.43	0.29–0.65		0.37	0.24–0.57	
1	0.55	0.38–0.80		0.56	0.38–0.84		0.53	0.35–0.79	
2	0.60	0.41–0.89		0.64	0.42–0.95		0.54	0.35–0.82	
3	Ref	Ref		Ref	Ref		Ref	Ref	
**Treatment protocol**			<0.001			<0.001			<0.001
dCRT + salvage surgery	0.95	0.67–1.33		0.76	0.51–1.13		0.76	0.49–1.18	
Upfront surgery	0.72	0.61–0.86		0.55	0.45–0.67		0.55	0.44–0.68	
Surgery + adjuvant CT	1.39	1.10–1.77		1.58	1.24–2.00		1.53	1.19–1.98	
nCRT + surgery	1.09	0.95–1.26		1.08	0.94–1.25		1.06	0.90–1.23	
nCT + surgery	Ref	Ref		Ref	Ref		Ref	Ref	
**Postoperative** complications*			<0.001			<0.001			<0.001
None or minor	0.57	0.50–0.64		0.77	0.68–0.88		0.74	0.64–0.86	
Major	Ref	Ref		Ref	Ref		Ref	Ref	

HR_adj_, adjusted hazard ratio; AC, adenocarcinoma; SCC, squamous cell carcinoma; (*n*)CT, (neoadjuvant)chemotherapy; (n/d)CRT, (neoadjuvant/definitive)chemoradiation; MIE, minimally invasive oesophagectomy; Ref, reference group. *Postoperative complications are graded according to the to the Clavien–Dindo scale^16^. Minor are considered as grade < IIIa and major as grade ≥ IIIa.

**Fig. 1:**
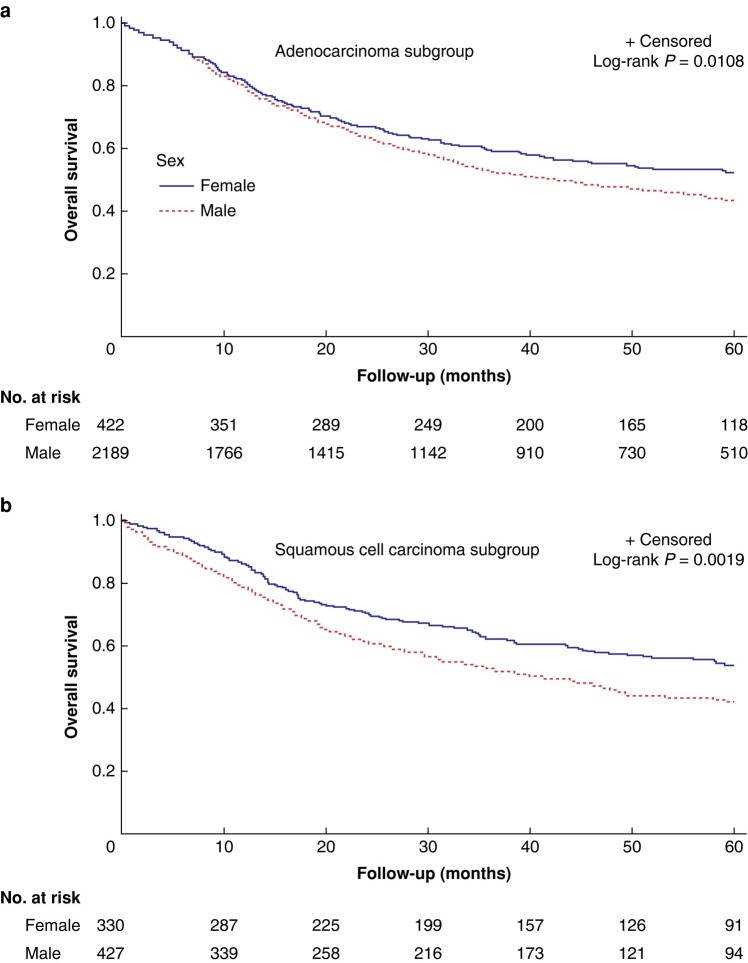
**Overall survival for male *versus* female patients in the ENSURE study**.

Within the AC subgroup, male patients had a median OS of 32.0 months (95% c.i. 12.9 to 58.8) *versus* 37.6 months (95% c.i. 14.8 to 60) for females (*P* = 0.011), whereas median OS in the SCC subgroup was 30.4 months (95% c.i. 12.2 to 56.1) in men *versus* 38.9 months (95% c.i. 15.8 to 60) in women (*P* = 0.002) (*Fig. 1*). Male patients with AC had poorer OS (adjusted HR 1.42, 95% c.i. 1.07 to 1.89) and DFS (adjusted HR 1.25, 95% c.i. 1.03 to 1.52); however, in the SCC group, no survival differences remained significant in multivariable analysis (*Table S7*).

**a** Adenocarcinoma: male patients had a median OS of 32.0 months (95% c.i. 12.9 to 58.8) *versus* 37.6 months (95% c.i. 14.8 to 60) for female patients (*P* = 0.011). **b** Squamous cell carcinoma: median OS in the SCC subgroup was 30.4 months (95% c.i. 12.2 to 56.1) in male patients *versus* 38.9 months (95% c.i. 15.8 to 60) in female patients respectively (*P* = 0.002). ENSURE, European iNvestigation of SUrveillance after Resection for Esophageal cancer; OS, overall survival; SCC, squamous cell carcinoma.

Recurrence patterns were similar in both sexes, with local recurrence observed in 18.3% male and 18.1% female patients, and distant recurrence in 33.1% and 26.7% respectively. Within histological subgroup analyses, no significant differences were observed in local or distant recurrence rates for patients with AC or SCC (*Table S5*).

### HRQoL after oesophagectomy

**Fig. 2:**
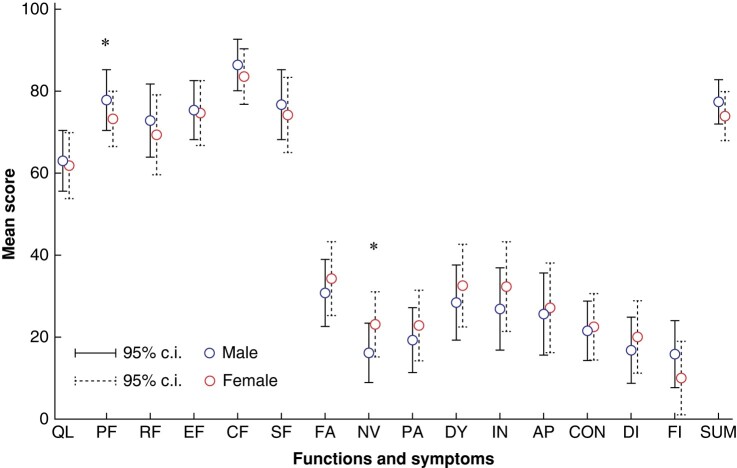
**Adjusted results of health-related quality of life (HRQoL) variables for male and female patients**.

*Figure 2* illustrates the adjusted mean scores of all QLQ-30 items. In multivariable linear regression, male patients had better scores of physical functioning (mean 79.1 (95% c.i. 72.9 to 85.3) *versus* 73.3 (95% c.i. 66.5 to 80.1) in female patients, *P* = 0.017) (detailed data in *Table S8*). Among specific symptoms, nausea-vomiting scores were higher in women (mean 23.1 (95% c.i. 15.2 to 31.1) *versus* 16.1 (95% c.i. 8.9 to 23.4) in men, *P* = 0.013). In multivariable linear regression, male sex remained independently associated with higher mean HRQoL summary scores (77.5 (95% c.i. 72.1 to 82.9) *versus* 72.6 (95% c.i. 66.7 to 78.6) in females, *P* = 0.022, *Table S9*). In this model, lower ASA class was also associated with higher HRQoL scores, whereas histological type, treatment protocol and surgical approach were not significant (*Table S9*). Within patients with AC, men presented higher physical functioning scores (mean 80.6 (95% c.i. 74.1 to 87.2) *versus* 73.8 (95% c.i. 65.8 to 81.9) in women, *P* = 0.023), but also more financial problems (mean score 9.7 (95% c.i. 1.1 to 18.2) *versus* 1.9 (95% c.i. −8.8 to 12.5), *P* = 0.049). In the SCC subgroup, female patients had higher scores of persistent diarrhoea (22.9 (95% c.i. 12.8 to 33.0) *versus* 11.9 (95% c.i. 1.2 to 22.7) in male patients, *P* = 0.043) (*Table S8*).

All variables are presented with mean score differences and 95% c.i. after adjusting for ASA class, sex, histological type, treatment protocol and surgical approach. Male patients had higher mean physical functioning scores (79.1 *versus* 73.3, *P* = 0.017), whereas female patients had higher nausea and vomiting symptom scores (23.1 *versus* 16.1, *P* = 0.013). Significant differences are illustrated with an asterisk (*). Absolute values of each score can be found in *Table S8*. QL, Global Quality of Life; PF, physical functioning; RF, role functioning; EF, emotional functioning; CF, cognitive functioning; SF, social functioning; FA, fatigue; NV, nausea-vomiting; PA, pain; DY, dyspnea; IN, insomnia; AP, appetite loss; CON, constipation; DI, diarrhoea; FI, financial problems; SUM, summary score.

## Discussion

In this large series drawn from the ENSURE study database, whose goal was to determine whether sex-related differences exist in oncologic, operative and HRQoL outcomes in the curative management of OC, several key findings emerged. First, female patients had improved long-term OS and DFS after curative treatment, especially in the AC subgroup. Second, although operative complication types, frequency and postoperative mortality rate were similar, more severe complications occurred in women. Finally, in terms of HRQoL in disease-free patients, men had higher functional and overall scores but also a higher risk of financial problems, while women had higher rates of persistent nausea and vomiting as well as diarrhoea in SCC.

There is a clear male predominance in OC, particularly AC, although the physiopathology remains unclear. Female sex hormones are purported to play a protective role against the malignant transformation process of AC^8,10,19^. Conversely, increased rates of SCC are documented in women, although exposure to alcohol and tobacco remains lower than in men^3,20–23^. Human papilloma virus (HPV)-16 exposure may also be carcinogenic, but the impact of sex in this regard is unknown^24^. Prior mediastinal radiation (e.g. for lymphoma, breast cancer) might also contribute to sex-related differences in SCC incidence; however, such data were unavailable for our analysis. In terms of management, a sex-related treatment gap has been reported^7,20^, with female patients less frequently receiving neoadjuvant treatment for locally advanced OC, and less systemic chemotherapy for metastatic disease^8^. In the present study, such treatment allocation differences could not be confirmed, neither in the neoadjuvant setting nor in the cancer-specific treatment of recurrence.

Overall, female patients presented with earlier disease stage (cN0) upon diagnosis, consistent with previously published studies^25^. The key finding of this study is an improved overall and disease-free survival in female patients, after similar treatments with curative intent. Although some authors previously found no sex-related survival differences in OC^23^, recent Swedish registry data revealed improved 5-year survival in female patients with SCC both after either curative surgery or dCRT^6,11,26^, which is also in accordance with reports from Japan^3,21^. The present study, including patients from a variety of European and North-American centres, highlights that survival was improved for women in the AC group, even after adjusting for confounders such as cN stage, ASA class and type of treatment modality. To explain these survival differences, a difference in treatment efficacy in AC might be assumed. However, the original CROSS (Chemoradiotherapy for Oesophageal Cancer Followed by Surgery Study) trial revealed a more pronounced treatment effect of nCRT in male patients compared with female patients^27^. In the present analysis, the predominant SCC subtype in females is thought to account for the better histologic response to neoadjuvant treatment, as no sex-related differences in ypTNM or TRG were observed within separate histological subtypes. As the baseline differences in cN0 stage and treatment modalities were adjusted for in all multivariable analyses, inherent biologic differences may also be suggested to explain outcome differences between male and female patients with OC, as previously described in gastric^7^ and colon cancer^9^. Indeed, oestrogen receptor β (ERβ) is strongly expressed on the nucleus of oesophageal AC cells^28,29^. When activated it entails an arrest of the cell cycle, leading to antiproliferative (tumour suppressor)activity^28^. Although the interplay between circulating sex hormones and hormone receptors in OC cancer cells could provide a pathophysiological basis to explain differences in tumour progression and survival, further research is needed to delve into this hypothesis.

In the present series, women had significantly higher rates of major postoperative complications. Previously, a large American cohort displayed higher rates of mortality and failure to rescue in female compared with male patients presenting with acute surgical pathologies of similar severity^30^. Potential sex-related disparities at presenting symptoms, time to diagnosis and aggressive care were suggested to explain this intriguing finding. In the present study, although the higher rates of severe complications observed in females might be partly attributed to the increased rates of open and salvage surgery, some further hypotheses can be made. Baseline performance status was similar and ASA class was even lower in female patients; however, these surrogate parameters may be misleading, as they do not accurately reflect sarcopenia and physiological reserves. In a series of patients with lung cancer, Rizzo *et al.* described higher rates of sarcopenia and muscle wasting in female patients compared with male patients of similar BMI; in turn, sarcopenia increases the risk of postoperative complications^31,32^. Thus, more precise screening methods are needed to determine physical status and conditioning in patients with OC, given their prognostic value on postoperative outcomes. The longer hospital stay observed in women may also be a consequence of a more severe postoperative morbidity rate in the overall cohort, though it may also reflect the potential loss of autonomy and unavailability of caregivers for patients undergoing oesophagectomy^20^. In several sociocultural contexts, female caregivers may be more readily available to take charge of a convalescent spouse/family member after oesophagectomy. However, as discharge criteria may vary among participating centres in our series, precise factors influencing duration of stay cannot be analysed in further detail.

With a reduction in operative mortality rate and improved survival rates, clinical research is increasingly focused on HRQoL. Previous data from the field of rectal cancer surgery demonstrated better functional outcomes, lower pain levels and less invalidating gastrointestinal symptoms in male patients 2 years after treatment^33^. The present analysis is the first, to the authors' knowledge, to report long-term HRQoL differences between male and female patients with OC. Although mean differences were not large per se and thus need to be interpreted with caution, patient sex remained independently associated with HRQoL outcomes in multivariable analysis, after adjusting for ASA class, histological type, treatment protocol and surgical approach. Female patients had higher rates of persistent gastrointestinal symptoms, while male patients presented better functional and overall HRQoL scores, resulting in a quicker return to everyday activity, but interestingly, also a higher risk of financial problems. Although caution is needed to not oversimplify a family model financially dependent on men’s income, we need to consider the risk of financial toxicity induced by OC and its management, especially in young patients^34^. These findings merit prospective assessment, allowing to incorporate within long-term follow-up the myriad nutritional, psychosocial and financial implications on OC and its therapy, and the differential challenges that sex and age may present.

The authors acknowledge some limitations in this study. The risk of confounding when assessing the main endpoints of the study has been addressed by performing separate subgroup analyses for each histological type, and multivariable analyses to adjust for the most clinically relevant confounding variables. However, there is still a risk that non-identified confounders may introduce bias to our results, with previously published studies reporting treatment allocation differences for OC patients even among European countries^35^. Namely, demographic parameters such as previous mediastinal radiation, dietary habits, HPV infection or oesophageal motor disorders are not available for our patient cohort. In addition, patient choices and rationale for initial treatment allocation (for example to dCRT *versus* neoadjuvant CRT in SCC patients), as well as the proportion of patients not proceeding to surgery after neoadjuvant treatment, are not reflected in our results. Similarly, precise data on dCRT regimens are unavailable in our dataset. As high-dose (>55 Gy) radiation is known to increase postoperative complications after salvage oesophagectomy^36^, these details might have provided some further insight into the differences in postoperative morbidity rate observed in our study. A prospective cohort study including extensive demographic, patient- and treatment-related data might limit this drawback, as well as the missing data issue, inherent to the retrospective design. As the database created for the ENSURE study^37^ includes a large panel of prospectively collected data, we strongly believe that robust statistical methodology allows meaningful conclusions that reflect current practice and outcomes in a Western-world OC population.

In conclusion, the present study illustrates that female patients display improved overall and disease-free survival after curative treatment of OC, although long-term overall HRQoL and functional recovery was found to be better in male patients. These data should encourage large-network prospective clinical and scientific research on the impact of sex on the key outcome measures of OC in treatment and survivorship.

## Collaborators

Hans Van Veer, Lieven Depypere, Willy Coosemans, Philippe Nafteux, Paul Carroll, Frances Allison, Gail Darling, John M. Findlay, Serenydd Everden, Nicholas D. Maynard, Arun Ariyarathenam, Grant Sanders, Shameen Jaunoo, Pritam Singh, Simon Parsons, John Saunders, Ravinder Vohra, Aaditya Sinha, Benjamin H.L. Tan, John G. Whiting, Piers R. Boshier, Sheraz R. Markar, Giovanni Zaninotto, George B. Hanna, Alexander W. Phillips, S. Michael Griffin, Robert C. Walker, Tim J. Underwood, Guillaume Piessen, Jorg Theisen, Hans Friess, Christiane J. Bruns, Wolfgang Schröder, Chris G. Collins, Oliver J. McAnena, Siobhan Rooney, Aoife Quinn, Conor Toale, Thomas J. Murphy, Jessie A. Elliott, Narayanasamy Ravi, Claire L. Donohoe, John V. Reynolds, Marco Scarpa, Romeo Bardini, Silvia Degasperi, Luca Saadeh, Carlo Castoro, Rita Alfieri, Eleonora Pinto, Genny Mattara, Marianne C Kalff, Suzanne S. Gisbertz, Mark I. van Berge Henegouwen, Sander J.M. van Hootegem, Sjoerd M. Lagarde, B. Feike Kingma, Lucas Goense, Jelle P. Ruurda, Richard van Hillegersberg, Raymond Kennedy, P. Declan Carey, Leanne Prodehl, Peter J. Lamb, Richard J.E. Skipworth, Mariagiulia Dal Cero, Manuel Pera, Biying Huang, Fredrik Klevebro, Magnus Nilsson, Asif Johar, Pernilla Lagergren, Gustav Linder, Magnus Sundbom, Styliani Mantziari, Markus Schäfer and Nicolas Demartines.

## Supplementary Material

zrae026_Supplementary_Data

## Data Availability

The primary data presented in the current work are not publicly available, but can be provided upon request to the corresponding author.
