# Supplementary material for: Sex-related differences in oncologic outcomes, operative complications and health-related quality of life after curative-intent oesophageal cancer treatment: multicentre retrospective analysis
Source: BJS Open. 2024 Apr 3;8(2):zrae026. doi: 10.1093/bjsopen/zrae026 (PMC10989878; doi:10.1093/bjsopen/zrae026)
Supplement: zrae026_Supplementary_Data [file zrae026_supplementary_data.docx]

**Sex-related differences in oncologic outcomes, operative complications, and health-related quality of life after curative-intent oesophageal cancer treatment: multicentre retrospective analysis**

Styliani Mantziari^1,2^, Jessie A Elliott^3^, Sheraz R Markar^4,5^, Fredrik Klevebro^6^, Lucas Goense^7^, Asif Johar^5^, Pernilla Lagergren^5,6^, Giovanni Zaninotto^8^, Richard van Hillegersberg^7^, Mark I van Berge Henegouwen^9,10^, Markus Schäfer^1,2^, Magnus Nilsson^5,6^, George B Hanna^8^, John V Reynolds^3^, for the ENSURE Study Group

1. Lausanne University Hospital CHUV, Switzerland

2. Faculty of Biology and Medicine, University of Lausanne UNIL, Switzerland

3.  Trinity St. James’s Cancer Institute, Trinity College Dublin, and St. James’s Hospital, Dublin, Ireland

4.    Surgical intervention trials Unit, Nuffield Department of Surgical Sciences, University of Oxford, UK

5.   Karolinska Institutet, Department of Molecular Medicine and Surgery, Karolinska University Hospital, Stockholm, Sweden

6.         CLINTEC, Karolinska Institutet, Stockholm, Sweden

7.  Department of Surgery, University Medical Center Utrecht, Utrecht University, Utrecht, the Netherlands

8. Department of Surgery and Cancer, Imperial College London, United Kingdom

9.         Department of Surgery, Amsterdam UMC, University of Amsterdam, the Netherlands

10. Cancer Center Amsterdam, the Netherlands

Correspondence to:

John V. Reynolds

Trinity St. James’s Cancer Institute, James Street, Dublin 8, D08NHY1 Ireland

Phone number: +353 18962189

Email: [reynoldsjv@stjames.ie](mailto:reynoldsjv@stjames.ie)

**Supplementary Materials - Index**

| **Supplementary Tables** |  |
| --- | --- |
| Table S1 Detailed list of the ENSURE study group centers. | *page 3* |
| Table S2 Clinicopathological characteristics for each histological subtype. | *page 4* |
| Table S3 Histopathologic characteristics stratified by sex, for each histological subtype. | *page 5* |
| Table S4 Postoperative outcomes for all patients. | *page 7* |
| Table S5 Treatment strategy and long-term recurrence for a. the entire cohort and b. each histological type separately.  Table S6 Baseline characteristics for male and female patients with upfront surgery and dCRT+ Salvage surgery.  Table S7 Cox regression results for Overall (OS), Disease-Free (DFS) and Disease-Specific (DSS) survival analyses, for each histological type.  Table S8 Adjusted HRQL summary score means for included variables in the multivariable linear regression model.  Table S9 Linear regression results for EORTC QLQ-30 functional and symptom scales for male and female patients. | *page 8*  *page 10*  *page 11*  *page 12*  *page 14* |
|  |  |

**Supplementary Figures and Tables**

**Table S1** Detailed list of the ENSURE study group centers.

| \| **Institution** \| **Country** \| \| --- \| --- \| \| University Hospital Leuven, Leuven \| Belgium \| \| University of Toronto \| Canada \| \| St. Mary's Hospital, London \| England \| \| Queen Elizabeth Hospital, Birmingham \| England \| \| Churchill Hospital, Oxford \| England \| \| Nottingham City Hospital \| England \| \| Southampton General Hospital \| England \| \| Centre Hospitalier Régional Universitaire de Lille \| France \| \| St. James's Hospital, Dublin \| Ireland \| \| Galway University Hospital, Ireland \| Ireland \| \| Mercy University Hospital, Cork \| Ireland \| \| Veneto Institute of Oncology, Padova \| Italy \| \| Azienda Ospedaliera di Padova \| Italy \| \| Erasmus MC, Rotterdam \| Netherlands \| \| Academic Medical Centre, Amsterdam \| Netherlands \| \| University Medical Centre, Utrecht \| Netherlands \| \| Royal Infirmary, Edinburgh \| Scotland \| \| Hospital Universitario Del Mar, Barcelona \| Spain \| \| Karolinska Instituet, Stockholm \| Sweden \| \| Lausanne University Hospital CHUV, Lausanne \| Switzerland \| |  |  |
| --- | --- | --- | --- | --- | --- | --- | --- | --- | --- | --- | --- | --- | --- | --- | --- | --- | --- | --- | --- | --- | --- | --- | --- | --- | --- | --- | --- | --- | --- | --- | --- | --- | --- | --- | --- | --- | --- | --- | --- | --- | --- | --- | --- | --- |

**Table S2** Clinicopathological characteristics for each histological subtype

| **ADENOCARCINOMA** | | | | **SQUAMOUS CELL CANCER** | | |
| --- | --- | --- | --- | --- | --- | --- |
|  | **Males**  **N=2389** | **Females**  **N=456** | **P-value** | **Males**  **N=467** | **Females**  **N=355** | **P-value** |
| Age at diagnosis, years | 64.5 [10] | 65.2 [10.6] | 0.205 | 63.7 [9.2] | 64.7 [9.5] | 0.123 |
| ASA class  I  II  III  Missing data | 624 (26.1)  1206 (50.5)  527 (22.1)  32 (1.3) | 140 (30.7)  226 (49.6)  82 (18.0)  8 (1.7) | 0.049 | 90 (19.3)  185 (39.6)  106 (22.7)  86 (18.4) | 111(31.3)  146 (41.1)  76 (21.4)  22 (6.2) | 0.014 |
| Tumor site  EGJ  Distal third  Middle third  Upper third  Missing data | 1166 (48.8)  982 (41.1)  77 (3.2)  9 (0.4)  155 (6.5) | 216 (47.4)  184 (40.4)  21 (4.6)  1 (0.2)  34 (7.4) | 0.459 | 69 (14.8)  178 (38.1)  159 (34.0)  22 (4.7)  39 (8.4) | 33 (9.3)  127 (35.8)  157 (44.2)  17 (4.8)  21 (5.9) | 0.015 |
| cT stage  0  1  2  3  4  Missing data | 22 (0.9)  231 (9.7)  414 (17.3)  1402 (58.7)  107 (4.5)  213 (8.9) | 4 (0.9)  50 (11.0)  83 (18.2)  258 (56.6)  15 (3.3)  46 (10.0) | 0.673 | 4 (0.9)  49 (10.5)  63 (13.5)  224 (48.0)  23 (4.9)  104 (22.2) | 6 (1.7)  35 (9.9)  75 (21.1)  176 (49.6)  16 (4.5)  47 (13.2) | 0.176 |
| cN stage  0  1  2  3  Missing data | 735 (30.8)  944 (39.5)  440 (18.4)  49 (2.1)  221 (9.2) | 180 (39.5)  153 (33.6)  67 (14.7)  13 (2.9)  43 (9.4) | 0.001 | 134 (28.7)  173 (37.0)  42 (9.0)  9 (1.9)  109 (23.3) | 127 (35.8)  135 (38.0)  38 (10.7)  6 (1.7)  49 (13.8) | 0.656 |
| cM1 stage  Missing data | 37 (1.5)  17 (0.7) | 3 (0.7)  3 (0.7) | 0.191 | 8 (1.7)  54 (11.6) | 8 (2.3)  9 (2.5) | 0.720 |

AC= Adenocarcinoma, SCC= Squamous cell cancer, ASA= American Society of Anesthesiologists, EGJ= Esophagogastric Junction

**Table S3** Histopathologic characteristics stratified by sex, for each histological subtype.

| **ADENOCARCINOMA** | | | | | **SQUAMOUS CELL CANCER** | | |
| --- | --- | --- | --- | --- | --- | --- | --- |
|  | **Males**  **N=2389** | **Females**  **N=456** | | **P-value** | **Males**  **N=467** | **Females**  **N=355** | **P-value** |
| (y)pT stage  0  Tis  1  2  3  4  Missing data | 178 (7.5)  23 (1)  483 (20.2)  350 (14.7)  1209 (50.6)  116 (4.9)  30 (1.3) | 42 (9.2)  10 (2.2)  104 (22.8)  67 (14.7)  207 (45.4)  23 (5.0)  3 (0.7) | 0.080 | | 90 (19.3)  8 (1.7)  91 (19.5)  68 (14.6)  175 (37.5)  26 (5.6)  9 (1.9) | 80 (22.5)  6 (1.7)  53 (14.9)  49 (13.8)  138 (38.9)  17 (4.8)  12 (3.4) | 0.577 |
| (y)pN stage  0  1  2  3  Missing data | 1047 (43.8)  519 (21.7)  493 (20.6)  280 (11.7)  50 (2.1) | 225 (49.3)  89 (19.5)  94 (20.6)  41 (9.0)  7 (1.5) | 0.113 | | 269 (57.6)  119 (25.5)  51 (10.9)  18 (3.9)  10 (2.1) | 223 (62.8)  72 (20.3)  35 (9.9)  14 (3.9)  11 (3.1) | 0.325 |
| (y)pM1 stage  Missing data | 55 (2.3)  28 (1.2) | 9 (2.0)  0 | 0.641 | | 9 (1.9)  4 (0.9) | 13 (3.7)  3 (0.8) | 0.127 |
| Differentiation  G0  G1  G2  G3  Signet-ring  Missing data | 203 (8.5)  193 (8.1)  677 (28.3)  709 (29.7)  47 (2.0)  560 (23.4) | 40 (8.8)  38 (8.3)  128 (28.1)  124 (27.2)  9 (2.0)  117 (25.7) | 0.958 | | 67 (14.3)  24 (5,1)  137 (29.3)  126 (27.0)  1 (0.2)  112 (24.0) | 60 (16.9)  26 (7.3)  93 (26.2)  81 (22.8)  1 (0.3)  94 (26.5) | 0.352 |
| Lymphatic invasion (L1)  L0  Missing data | 700 (29.3)  1217 (50.9)  472 (19.8) | 123 (27.0)  239 (52.4)  94 (20.6) | 0.357 | | 99 (21.2)  238 (51.0)  130 (27.8) | 68 (19.2)  212 (59.7)  75 (21.1) | 0.157 |
| Venous invasion (V1)  V0  Missing data | 678 (28.4)  1309 (54.8)  402 (16.8) | 116 (25.4)  258 (56.6)  82 (18.0) | 0.243 | | 135 (28.9)  282 (60.4)  50 (10.7) | 82 (23.1)  218 (61.4)  55 (15.5) | 0.147 |
| Perineural invasion (Pn1)  Pn0  Missing data | 478 (20.0)  1268 (53.1)  643 (26.9) | 66 (14.5)  251 (55.0)  139 (30.5) | 0.015 | | 98 (21.0)  291 (62.3)  78 (16.7) | 44 (12.4)  216 (60.8)  95 (26.8) | 0.013 |
| Resection margin (R) status  0  1  2  Missing data | 2032 (85.1)  323 (13.5)  4 (0.2)  30 (1.3) | 379 (83.1)  70 (15.4)  1 (0.2)  6 (1.3) | 0.560 | | 404 (86.5)  56 (12.0)  4 (0.9)  3 (0.6) | 316 (¢9.0)  33 (9.3)  2 (0.6)  4 (1.1) | 0.421 |
| Mandard regression grade*  TRG 1  TRG 2  TRG 3  TRG 4  TRG 5  Missing data | 155 (6.5)  191 (8.0)  228 (9.5)  379 (15.9)  209 (8.7)  503 (21.1) | 27 (5.9)  31 (6.8)  46 (10.1)  68 (14.9)  35 (7.7)  76 (16.7) | 0.179 | | 59 (12.6)  33 (7.1)  29 (6.2)  29 (6.2)  16 (3.4)  146 (31.3) | 56 (15.8)  27 (7.6)  25 (7.0)  25 (7.0)  18 (5.1)  87 (24.5) | 0.857 |
| Lymph nodes involved | 2.8 [ 4.7] | 2.3 [4.2] | 0.008 | | 1.4 [3.1] | 1.3 [2.8] | 0.062 |
| Lymph nodes analyzed | 25.4 [11.4] | 25.5 [12.1] | 0.069 | | 31.9 [22.5] | 25.9 [14.6] | 0.001 |

Continuous variables are expressed as mean [SD] and categorical as n (%).

*TRG=Tumor Regression Grade (only available in patients that underwent neoadjuvant chemotherapy)

**Table S4** Postoperative outcomes for all patients

|  | All  N=3974 | Male  N=3083 | Female  N=891 | P-value |
| --- | --- | --- | --- | --- |
| Length of stay,days | 20.7 [21.5] | 20.2 [20.9] | 22.7 [23] | 0.005 |
| Critical care length of stay, days | 6.9 [14.3] | 6.9 [14.4] | 6.8 [13.9] | 0.931 |
| Comprehensive complications Index (CCI)* | 20.3 [23.3] | 20.2 [23.9] | 20.7 [21.6] | 0.709 |
| Postoperative morbidity  Missing data | 2464 (62)  143 (3.6) | 1898 (61.6)  112 (3.6) | 566 (63.6)  31 (3.5) | 0.298 |
| Postoperative complications  No complications  Minor (Clavien <IIIa)  Major (Clavien ≥IIIa) | 1441 (36.3)  1204 (30.3)  1260 (31.7) | 1129 (36.6)  949 (30.8)  949 (30.8) | 312 (35.0)  255 (28.6)  311 (34.9) | 0.023 |
| *AC group* major compl. |  | 669 (28.0) | 145 (31.8) | 0.108 |
| *SCC group* major compl.  Missing data | 69 (1.7) | 197 (42.2)  56 (1.8) | 139 (39.2)  13 (1.5) | 0.359 |
| Pulmonary complications | 1419 (35.7) | 1097 (35.6) | 322 (36.1) | 0.502 |
| Anastomotic leak** | 388 (9.8) | 298 (9.7) | 90 (10.1) | 0.703 |
| In-hospital mortality | 140 (3.5) | 111 (3.6) | 29 (3.3) | 0.611 |

Discrete variables are shown as n (%), whereas continuous ones as mean [SD]

CCI= Comprehensive Complications Index, AC= Adenocarcinoma, SCC= Squamous cell carcinoma, Compl.= complications

**As per Slankamenac K, Nederlof N, Pessaux P, et al. Ann Surg 2014.Nov; 260 (5): 757-62*

*** As per Low DE, Alderson D, Ceconnello I, et al. Ann Surg 2015.272: 286-294.*

**Table S5.** Treatment strategy and long-term recurrence for a. the entire cohort and b. each histological type separately

| 1. **Entire cohort** | **All** | **Male** | **Female** | **P-value** |
| --- | --- | --- | --- | --- |
|  | **N= 3974** | **N=3083** | **N=891** |  |
| Neoadjuvant treatment | 2324 (58.5) | 1824 (59.2) | 500 (56.2) | 0.104 |
| Neoadjuvant regimen  MAGIC CT  FLOT CT  Other CT  CROSS CRT  Other CRT | 916 (23.0)  4 (0.1)  541 (13.6)  390 (9.8)  506 (12.7) | 731 (23.7)  2 (0.1)  416 (13.5)  303 (9.8)  399 (12.9) | 185 (20.8)  2 (0.2)  125 (14.0)  87 (9.8)  107 (12) | 0.425 |
| Treatment protocol  Surgery only  Surgery+ adjuvant CT  nCT+ Surgery  nCRT+ Surgery  dCRT+ Salvage Surgery  Missing data | 815 (20.5)  136 (3.4)  1413 (35.6)  911 (22.9)  76 (1.9)  623 (15.7) | 595 (19.3)  109 (3.5)  1107 (35.9)  717 (23.3)  63 (2.0)  492 (16.0) | 220 (24.7)  27 (3.0)  306 (34.3)  194 (21.8)  13 (1.5)  131(14.7) | 0.014 |
| Type of Surgery  Extended gastrectomy  Ivor Lewis  McKeown  Transhiatal  Sweet/cervical anast.  Sweet/intrathoracic anast.  Missing data | 278 (7.0)  1886 (47.5)  702 (17.7)  219 (5.5)  653 (16.4)  100 (2.5)  136 (3.4) | 213 (6.9)  1513 (49.1)  504 (16.3)  155 (5.0)  521 (16.9)  76 (2.5)  101 (3.3) | 65 (7.3)  373 (41.9)  198 (22.2)  64 (7.2)  132 (14.8)  24 (2.7)  35 (3.9) | <0.001 |
| Surgical approach  Open  Hybrid MIE  Total MIE  Missing data | 2664 (67)  641 (16.1)  581 (14.6)  88 (2.2) | 2043 (66.3)  525 (17.0)  447 (14.5)  68 (2.2) | 621 (69.7)  116 (13.0)  134 (15.0)  20 (2.2) | 0.016 |
| Long-term recurrence  No recurrence  Local  Systemic  Missing data | 840 (21.1)  725 (18.2)  1258 (31.7)  1151 (29.0) | 694 (22.5)  564 (18.3)  1020 (33.1)  805 (26.1) | 146 (16.4)  161 (18.1)  238 (26.7)  346 (38.8) | 0.159 |

| **B. By histology ADENOCARCINOMA** | | | | | **SQUAMOUS CELL CANCER** | | | | |
| --- | --- | --- | --- | --- | --- | --- | --- | --- | --- |
|  | **Males**  **N=2389** | **Females**  **N=456** | **P-value** | **Males**  **N=467** | | **Females**  **N=355** | | **P-value** |  |
| Neoadjuvant treatment | 1432 (59.9) | 264 (57.9) | 0.414 | | 268 (57.4) | | 198 (55.8) | | 0.644 |
| Treatment protocol  Surgery only  Surgery+ Adjuvant CT  nCT+ Surgery  nCRT+ Surgery  dCRT+Salvage Surgery | 458 (19.2)  84 (3.5)  940 (39.3)  492 (20.6)  23 (1.0) | 115 (25.2)  14 (3.1)  193 (42.3)  71 (15.6)  2 (0.4) | 0.009 | | 84 (18.0)  15 (3.2)  124 (26.6)  144 (30.8)  12 (2.6) | | 80 (22.5)  9 (2.5)  92 (25.9)  106 (29.9)  6 (1.7) | | 0.531 |
| Missing data | 392 (16.4) | 61 (13.4) |  | | 88 (18.8) | | 62 (17.5) | |  |
| Type of Surgery  Extended gastrectomy  Ivor Lewis  McKeown  Transhiatal  Sweet/cervical anast.  Sweet/intrathoracic anast. | 204 (8.5)  1204 (50.4)  309 (12.9)  135 (5.7)  460 (19.3)  67 (2.8) | 61 (13.4)  195 (42.8)  59 (12.9)  43 (9.4)  82 (18.0)  15 (3.3) | <0.001 | | 5 (1.1)  147 (31.5)  151 (32.3)  17 (3.6)  55 (11.8)  7 (1.5) | | 2 (0.6)  127 (35.8)  128 (36.1)  19 (5.3)  45 (12.7)  8 (2.2) | | 0.866 |
| Missing data | 10 (0.4) | 1 (0.2) |  | | 85 (18.2) | | 26 (7.3) | |  |
| Approach  Open  Hybrid MIE  Total MIE | 1590 (66.6)  385 (16.1)  368 (15.4) | 315 (69.1)  55 (12.0)  77 (16.9) | 0.083 | | 327 (70.0)  6 (1.3)  71 (15.2) | | 263 (74.1)  33 (9.3)  52 (14.6) | | 0.222 |
| Missing data | 46 (1.9) | 9 (2.0) |  | | 63 (13.5) | | 7 (2.0) | |  |
| Length of stay (days) | 19.0 [20] | 22.3 [26] | 0.014 | | 25.2 [25] | | 23.6 [20.3] | | 0.338 |
| Long-term recurrence  No recurrence  Local  Systemic  Missing data | 563 (23.6)  418 (17.5)  820 (34.3)  588 (24.6) | 73 (16.0)  74 (16.2)  122 (26.8)  187 (41.0) | 0.063  0.951 | | 76 (16.3)  86 (18.4)  117 (25.1)  188 (40.2) | | 51 (14.4)  74 (20.8)  87 (24.5)  143 (40.3) | | 0.334  0.832 |

Continuous variables are expressed as mean [SD], whereas categorical as n (%).

(n)CT= (neoadjuvant)Chemotherapy, (n/d) CRT= (neoadjuvant/definitive) Chemoradiation, MIE= Minimally Invasive Esophagectomy

**Table S6**. Baseline characteristics for male and female patients with upfront surgery and dCRT+ Salvage surgery

|  | 1. **Upfront surgery** | | | 1. **dCRT+ Salvage surgery** | | |
| --- | --- | --- | --- | --- | --- | --- |
|  | Males  N=815 | Females  N=595 | *P*-value | Males  N=76 | Females  N=63 | *P*-value |
| Age, years | 66.8 [19.7] | 67.0 [11.1] | 0.827 | 61 [8.7] | 62 [7.5] | 0.714 |
| ASA class  I  II  III | 147 (21.9)  339 (50.5)  186 (27.7) | 65 (27.8)  121 (51.7)  48 (20.5) | 0.048 | 5 (7.9)  31 (49.2)  27 (42.9) | 1 (7.7)  10 (76.9)  2 (15.4) | 0.159* |
| ECOG status  0  1  2  3 | 318 (55.4)  198 (34.5)  47 (8.2)  11 (1.9) | 120 (60.6)  61 (30.8)  15 (7.6)  2 (1.0) | 0.555 | 31 (53.5)  25 (43.1)  1 (1.7)  1 (1.7) | 6 (50)  5 (41.7)  1 (8.3)  0 (0) | 0.549 |
| cT stage  0  1  2  3  4 | 20 (3.3)  212 (34.8)  170 (27.9)  197 (32.3)  11 (1.8) | 10 (4.8)  67 (32.2)  62 (29.8)  65 (31.2)  4 (1.9) | 0.817 | 0 (0)  6 (10)  16 (26.7)  37 (61.7)  1 (1.7) | 0 (0)  2 (15.4)  3 (23.1)  7 (53.9)  1 (7.8) | 0.503* |
| cN stage  0  1  2  3 | 343 (56.7)  199 (32.9)  53 (8.8)  10 (1.7) | 139 (66.5)  57 (27.3)  12 (5.7)  1 (0.5) | 0.059 | 9 (14.8)  22 (36.1)  30 (49.2)  0 (0) | 1 (7.7)  5 (38.5)  7 (53.9)  0 (0) | 0.919* |

Continuous variables are expressed as mean [SD], whereas categorical as n (%).

*Fisher’s exact test. ECOG= Eastern Cooperative Status Scale, ASA= American Society of Anesthesiologists.

**Table S7.** Cox regression results for Overall (OS), Disease-Free (DFS) and Disease-Specific (DSS) survival analyses, for each histological type.

|  | Unadjusted HR | 95%CI | Adjusted HR | 95% CI |
| --- | --- | --- | --- | --- |
| i. OVERALL SURVIVAL (OS) |  |  |  |  |
| AC |  |  |  |  |
| Male sex | 1.22 | 1.03-1.44 | 1.42 | 1.07-1.89 |
| SCC |  |  |  |  |
| Male sex | 1.51 | 1.16-1.98 | 1.17 | 0.97-1.40 |

| ii. DISEASE-FREE SURVIVAL (DFS) |  |  |  |  |
| --- | --- | --- | --- | --- |
| AC |  |  |  |  |
| Male sex | 1.37 | 1.14-1.64 | 1.25 | 1.03-1.52 |
| SCC |  |  |  |  |
| Male sex | 1.25 | 0.93-1.68 | 1.17 | 0.86-1.60 |

| iii. DISEASE-SPECIFIC SURVIVAL (DSS) |  |  |  |  |
| --- | --- | --- | --- | --- |
| AC |  |  |  |  |
| Male sex | 1.28 | 1.05-1.55 | 1.16 | 0.94-1.43 |
| SCC |  |  |  |  |
| Male sex | 1.31 | 0.95-1.82 | 1.25 | 0.89-1.77 |

AC= adenocarcinoma, SCC= squamous cell cancer, HR= hazard ratio, 95% CI= 95% Confidence Intervals, nCRT= neoadjuvant chemoradiation, nCT= neoadjuvant chemotherapy, dCRT= definitive chemoradiation

*All multivariable analyses are adjusted for age, sex, histological type, cN stage, ASA class, type of treatment protocol (e.x neoadjuvant treatment and surgery, definitive chemoradiation and salvage surgery, upfront surgery) and occurrence of major postoperative complications (Clavien ≥IIIa)

Female sex was the reference group (HR=1) in all multivariable models.

**Table S8**. Linear regression results for EORTC QLQ-30 functional and symptom scales for male and female patients

| 1. All patients | Males |  | Females |  | *P*-value |
| --- | --- | --- | --- | --- | --- |
|  | Least square mean | 95% CI | Least square mean | 95% CI |  |
| **Physical Functioning (pf)** | **79.1** | **72.9-85.3** | **73.3** | **66.5-80.1** | **0.017** |
| Role functioning (rf) | 72.8 | 63.9-81.8 | 69.4 | 59.7-79.2 | 0.323 |
| Emotional functioning (ef) | 75.4 | 68.2-82.6 | 74.7 | 66.8-82.6 | 0.797 |
| Cognitive functioning (cf) | 86.4 | 80.2-92.7 | 83.6 | 76.7-90.4 | 0.235 |
| Social functioning (sf) | 76.8 | 68.4-85.3 | 74.3 | 65.1-83.6 | 0.444 |
| Global health status (QoL) | 63.1 | 55.7-70.5 | 62.0 | 53.9-70.1 | 0.703 |
| Fatigue | 30.7 | 22.5-39.0 | 34.2 | 25.2-43.2 | 0.274 |
| **Nausea-Vomiting** | **16.1** | **8.9-23.4** | **23.1** | **15.2-31.1** | **0.013** |
| Pain | 19.3 | 11.3-27.2 | 22.9 | 14.2-31.6 | 0.240 |
| Dyspnoea | 28.4 | 19.3-37.6 | 32.6 | 22.5-42.6 | 0.245 |
| Insomnia | 26.9 | 16.9-36.9 | 32.3 | 21.4-43.2 | 0.164 |
| Appetite loss | 25.6 | 15.5-35.6 | 27.1 | 16.2-38.1 | 0.687 |
| Constipation | 21.5 | 14.2-28.8 | 22.6 | 14.6-30.6 | 0.701 |
| Diarrhea | 16.8 | 8.7-24.9 | 20.0 | 11.2-28.9 | 0.302 |
| Financial problems | 15.8 | 7.7-23.9 | 10.0 | 1.1-19.9 | 0.069 |
| Summary score | 77.4 | 71.9-82.8 | 73.8 | 67.9-79.6 | 0.094 |

| 1. AC | Least square mean | 95% CI | Least square mean | 95% CI | P-value |
| --- | --- | --- | --- | --- | --- |
| **Physical Functioning (pf)** | **80.6** | **74.1-87.2** | **73.8** | **65.8-81.9** | **0.023** |
| Role functioning (rf) | 68.8 | 59.2-78.3 | 67.2 | 55.5-79.0 | 0.726 |
| Emotional functioning (ef) | 75.6 | 68.1-83.1 | 78.7 | 69.4-87.9 | 0.365 |
| Cognitive functioning (cf) | 82.9 | 76.4-89.5 | 80.6 | 72.6-88.7 | 0.436 |
| Social functioning (sf) | 75.5 | 66.5-84.4 | 74.6 | 63.6-85.7 | 0.842 |
| Global health status (QoL) | 60.5 | 52.6-68.4 | 61.3 | 51.6-71.0 | 0.819 |
| Fatigue | 34.5 | 25.8-43.2 | 38.8 | 28.2-49.5 | 0.273 |
| Nausea-Vomiting | 12.4 | 4.8-19.9 | 18.0 | 8.7-27.4 | 0.103 |
| Pain | 23.8 | 15.4-32.3 | 27.5 | 17.1-37.9 | 0.339 |
| Dyspnoea | 21.2 | 11.7-30.7 | 24.2 | 12.5-35.9 | 0.492 |
| Insomnia | 27.5 | 17.0-37.9 | 29.8 | 16.9-42.7 | 0.621 |
| Appetite loss | 30.1 | 19.5-40.8 | 33.1 | 20.0-46.2 | 0.541 |
| Constipation | 13.3 | 5.9-20.8 | 14.5 | 5.3-23.7 | 0.727 |
| Diarrhea | 16.0 | 7.5-24.5 | 14.8 | 4.4-25.2 | 0.756 |
| **Financial problems** | **9.7** | **1.1-18.2** | **1.9** | **-8.8,12.5** | **0.049** |
| Summary score | 77.2 | 71.5-82.9 | 74.8 | 67.7-81.9 | 0.354 |

| 1. SCC | Least square mean | 95% CI | Least square mean | 95% CI | P-value |
| --- | --- | --- | --- | --- | --- |
| Physical Functioning (pf) | 79.4 | 71.1-87.7 | 73.9 | 66.1-81.8 | 0.190 |
| Role functioning (rf) | 70.6 | 58.5-82.7 | 62.9 | 51.5-74.3 | 0.204 |
| Emotional functioning (ef) | 79.9 | 70.4-89.5 | 71.7 | 62.7-80.7 | 0.087 |
| Cognitive functioning (cf) | 85.9 | 77.5-94.2 | 82.7 | 74.9-90.6 | 0.449 |
| Social functioning (sf) | 80.6 | 69.2-91.9 | 73.2 | 62.4-83.9 | 0.193 |
| Global health status (QoL) | 60.9 | 50.9-70.9 | 56.3 | 46.9-65.7 | 0.357 |
| Fatigue | 36.8 | 25.8-47.8 | 37.7 | 27.4-48.1 | 0.864 |
| Nausea-Vomiting | 13.7 | 4.1-23.3 | 22.6 | 13.5-31.7 | 0.066 |
| Pain | 24.1 | 13.3-34.8 | 27.3 | 17.2-37.4 | 0.548 |
| Dyspnoea | 17.6 | 5.5-29.6 | 25.1 | 13.7-36.4 | 0.215 |
| Insomnia | 25.3 | 12.0-38.5 | 33.5 | 20.9-45.9 | 0.219 |
| Appetite loss | 33.8 | 20.3-47.3 | 33.6 | 20.9-46.3 | 0.974 |
| Constipation | 13.6 | 4.1-23.0 | 15.1 | 6.2-24.1 | 0.742 |
| **Diarrhea** | **11.9** | **1.2-22.7** | **22.9** | **12.8-33.0** | **0.043** |
| Financial problems | 11.7 | 0.9-22.6 | 10.6 | 0.3-20.9 | 0.837 |
| Summary score | 78.5 | 71.2-85.7 | 72.8 | 66.0-79.7 | 0.122 |

AC= Adenocarcinoma, SCC= Squamous Cell Carcinoma, 95% CI= 95% Confidence intervals

**Table S9.** Adjusted HRQL summary score means for included variables in the multivariable linear regression model.

|  | Adjusted mean | 95% CI | *P*-value |
| --- | --- | --- | --- |
| ASA class  I  II  III | 78.7  76.3  70.2 | 72.4-84.9  71.1-81.6  63.7-76.6 | 0.003 |
| Gender  Male  Female | 77.5  72.6 | 72.1-82.9  66.7-78.6 | 0.022 |
| Histologic type  AC  Other  SCC | 75.4  73.7  76.1 | 70.2-80.6  64.0-83.3  70.4-81-8 | 0.759 |
| Treatment protocol  dCRT+Salvage surgery  Upfront surgery  Surgery + adjuvant CT  nCRT+ Surgery  nCT+ Surgery | 69.0  76.3  73.1  79.9  77.1 | 50.9-87.1  71.8-80.7  60.8-85.4  75.6-84.2  72.3-81.8 | 0.084 |
| Surgical approach  Open  Hybrid MIE  Total MIE | 76.3  74.3  74.7 | 68.0-80.5  70.9-81.6  68.0-81.3 | 0.409 |

All variables are presented as adjusted means (95%CI) from the generalized linear model (GLM), for the outcome variable HRQL Summary Score.

HRQL= Health-related Quality of Life, ASA= American Society of Anesthesiologists, AC= Adenocarcinoma, SCC= Squamous Cell carcinoma, (n)CT= (neoadjuvant) Chemotherapy, (n/d) CRT= (neoadjuvant/definitive) Chemoradiation, MIE= Minimally Invasive Esophagectomy
